# Comprehensive Analysis of the Prognostic Value of Circulating MMP-7 Levels in Urothelial Carcinoma: A Combined Cohort Analysis, Systematic Review, and Meta-Analysis

**DOI:** 10.3390/ijms24097859

**Published:** 2023-04-26

**Authors:** András Kubik, Isabel Pinto Amorim das Virgens, Anett Szabó, Melinda Váradi, Anita Csizmarik, Attila Keszthelyi, Attila Majoros, Péter Fehérvári, Péter Hegyi, Nándor Ács, Péter Nyirády, Tibor Szarvas

**Affiliations:** 1 Department of Urology, Semmelweis University, 1082 Budapest, Hungary; 2 Center for Translational Medicine, Semmelweis University, 1082 Budapest, Hungary; 3 Department of Biostatistics, University of Veterinary Medicine, 1078 Budapest, Hungary; 4 Institute for Translational Medicine, Medical School, University of Pécs, 7624 Pécs, Hungary; 5 Division of Pancreatic Diseases, Heart and Vascular Center, Semmelweis University, 1083 Budapest, Hungary; 6 Department of Obstetrics and Gynecology, Semmelweis University, 1088 Budapest, Hungary; 7 Department of Urology, University of Duisburg-Essen and German Cancer Consortium (DKTK), 45147 Essen, Germany

**Keywords:** urothelial cancer, urinary bladder cancer, MMP-7, matrilysin, biomarker, prognostication, radical cystectomy, lymphadenectomy, micrometastases, overall survival

## Abstract

Lymph node (LN) status is the most significant prognostic factor for invasive urothelial bladder cancer (UBC); however, the optimal extent of LN dissection (LND) is debated. We assessed circulating matrix metalloproteinase-7 (MMP-7) as a prognostic factor and decision-making marker for the extent of LND. Preoperative serum MMP-7 levels were determined in two independent UBC cohorts (n = 188; n = 68) and in one control cohort (n = 97) by using the ELISA method. A systematic review and meta-analysis on the prognostic role of circulating pretreatment MMP-7 levels were performed. Serum MMP-7 levels were higher in patients compared to controls (*p* < 0.001) with the highest levels in LN-positive cases. Half of LN-positive UBC patients had low MMP-7 levels, whereas the survival of LN-negative patients with high serum MMP-7 findings was poor. MMP-7 levels were independently associated with poor survival in both cohorts (*p* = 0.006, *p* < 0.001). Accordingly, our systematic review of six eligible publications revealed a 2.5-fold higher mortality risk in patients with high MMP-7 levels. In conclusion, preoperative MMP-7 level is a validated and independent prognostic factor in urothelial cancer. It cannot be used to decide between regional or extended LND but may be useful in identifying LN-negative high-risk patients with potentially undetected metastases.

## 1. Introduction

Urinary bladder cancer (UBC) is the second most common malignancy of the urinary tract, causing 572,000 new cases and more than 212,000 deaths worldwide each year [1]. Approximately 10–15% of patients are diagnosed with muscle-invasive bladder cancer (MIBC), and nearly 10% of newly diagnosed patients are diagnosed with locally advanced and/or metastatic disease. For these patients, perioperative chemotherapy (CTX), radical cystectomy (RC) with pelvic lymph node dissection (LND), and urinary diversion are the gold standard treatments [2]. Half of these patients benefit from radical surgery, while the other half of the patients progress rapidly and die within two years [3]. For these patients, lymph node (LN) positivity is the most significant risk factor [4], occurring in approximately 20–25% of RC-treated UBC patients [5,6]. However, LN positivity is difficult to detect prior to surgery, as detection of LN metastases is limited by the low sensitivity of imaging methods, especially in small metastases [7].

LN metastases have proved to be the most reliable prognostic factor for survival in patients undergoing RC [8]. The optimal extent of LND has been investigated in large prospective studies with conflicting results. Some of these studies showed that extended LND (also including the deep obturator, common iliac, presacral, paracaval, interaortocaval, and para-aortal nodes to the inferior mesenteric artery) may provide improved recurrence-free (RFS) and disease-specific survival (DSS) compared to limited LND (including only the obturator, and internal and external iliac nodes) [9,10]. In contrast, other studies have not found an association between the LND extent and patient outcome [11,12]. This suggests that performing extended LND may improve prognosis only in a subgroup of RC-treated patients [13,14]. Determining the odds of LN metastases preoperatively may help to select patients who will most likely benefit from extended LND and early systemic treatment.

Matrix metalloproteinase-7 (MMP-7) is the smallest member of the matrix metalloproteinase family with broad substrate specificity. Therefore, MMP-7 is involved in several tumor-supporting cellular processes, such as apoptosis, angiogenesis, and tumor-related osteolysis [15]. In UBC, elevated tissue, serum, and urine MMP-7 levels were found to be associated with the presence of LN metastases and an unfavorable prognosis of patients [16]. In this study, we aimed to further evaluate the prognostic relevance of preoperative serum MMP-7 levels in two independent UBC cohorts. Furthermore, we also assessed two additional new aspects of the potential role of serum MMP-7 levels in limited versus extended LND decision making, as well as therapeutic monitoring. Finally, we performed a systematic review and meta-analysis to provide a comprehensive overview of the clinical value of circulating MMP-7 levels in urothelial carcinoma.

## 2. Material and Methods

We performed a post hoc serum MMP-7 analysis in two independent patient cohorts, with a total of 256 UBC patients. We also performed a systematic review and meta-analysis of the literature on the prognostic value of pretreatment circulating MMP-7 levels in patients with urothelial carcinomas. The study was reported according to the Preferred Reporting Items for Systematic Reviews and Meta-analyses (PRISMA) 2020 recommendations [17] (Supplementary Table S1), and the Cochrane Handbook was followed [18]. The protocol was registered on PROSPERO (Nr. CRD42022367152).

### 2.1. Cohorts

For cohort 1, pretreatment serum samples were available for 188 UBC patients (137 males and 51 females) who underwent radical cystectomy (RC) (n = 87) or transurethral resection of the bladder (TURB) (n = 101) at the Department of Urology, University Hospital of Essen, Germany, between August 2008 and November 2013. 

Cohort 2 included 68 UBC patients (43 males and 25 females) with pretreatment serum samples who underwent radical cystectomy (RC) at the Department of Urology, Semmelweis University, Budapest, Hungary, between January 2014 and December 2018. Follow-up samples collected on postoperative days 2 and 5 were available for 57 and 46 patients, respectively. Additionally, for this cohort, LNs removed during radical surgery were separately collected and evaluated S1), and LND was performed according to the anatomic template defined by Gschwend et al. [11]. LNs were separated according to their locations, thus determining the extent of LN removal. Accordingly, LNs belonging to the limited versus extended LND were evaluated separately.

The study was conducted according to the ethical standards of the Declaration of Helsinki, and the study protocol was approved by the ethics committees of the University of Duisburg-Essen (15-6400-BO) and Semmelweis University (TUKEB 256/2014). The primary endpoint of this study was overall survival (OS). Time to death or survival was considered the time from the surgery (RC or TURB) to the relevant endpoint (death).

### 2.2. Serum MMP-7 ELISA Analysis

We used the enzyme-linked immunosorbent assay (ELISA) to determine serum MMP-7 levels using the Human Total MMP-7 Quantikine ELISA kit (R&D Systems, Wiesbaden, Germany, Catalog Number: DMP700), according to the product instructions. Colorimetric detection was performed by a Thermo Scientific™ Multiscan FC Microplate Photometer. The results were analyzed with the help of Skanlt 5.0 software. For the data set achieved by ELISA, the cut-off of 7.15 ng/mL serum MMP-7 level reported in a previously published study was used [16].

### 2.3. Meta-Analysis

#### 2.3.1. Literature Search

Two independent authors (AKu and IPAV) performed the systematic search and the selection process. Cohen’s kappa coefficient (κ) was calculated after each step to measure inter-rater reliability [19]. Electronic databases of PubMed, Scopus, EMBASE, the Cochrane Library, and Web of Science were searched to identify studies investigating the prognostic role of MMP-7 in urothelial cancer patients treated with radical surgery or systemic therapy (Text S1). Duplicates were removed using EndNote 20 (Clarivate Analytics, Philadelphia, PA, USA), and the remaining articles were assessed by title-abstract (κ = 0.92) and full text (κ = 1.0) using Rayyan [20].

#### 2.3.2. Eligibility Criteria

The PECO framework was applied to formulate our research question. We included original studies that investigated (P) urothelial cancer patients treated with surgery or systemic therapy and (E and C) compared the hazards of pretreatment high and low serum or plasma MMP-7 levels in terms of (O) overall survival. To define high and low levels of MMP-7, we used the included cut-off values for pretreatment serum and plasma biomarker levels at the protein level (e.g., via immunoassay) reported in each eligible article. We excluded studies with tissue biomarker levels, postoperative MMP-7 levels only, reviews, meta-analyses, conference abstracts, case reports, and case series. No restrictions were made based on cohort size or study design.

#### 2.3.3. Data Extraction

Two independent authors (AKu, IPAV) extracted data from articles. Data of interest included the name of the first author, year of publication, cancer type, stage, therapy received, country of sample/data collection, study type, cohort size, age and sex of patients, cut-off values for MMP-7 levels, method of determining cut-off values (e.g., median), assay method, follow-up time, OS, and cancer-specific survival (CSS). In the MIBC cohorts, the main outcome was OS, with the exception of one study [21], and was assessed as OS in the meta-analysis given the high mortality of the disease.

#### 2.3.4. Quality Assessment and Evaluation of Evidence

The risk of bias was assessed using the Quality in Prognostic Studies (QUIPS) tool [22] by two independent authors (Aku, IPAV). The RobVisR tool was used to summarize the results of the assessments [23] (Supplementary Table S2 and Figure S2).

#### 2.3.5. Synthesis Methods

Random-effects models with the inverse variance method were applied to pool log-transformed hazard ratios (HR) with a 95% confidence interval (CI). The restricted maximum-likelihood method [24] was used to estimate variance measure τ^2^, and between-study heterogeneity was investigated with the Cochrane Q test and Higgins and Thompson’s I^2^ statistics [25]. Forest plots were used to graphically summarize the results. Where applicable, we reported the prediction intervals of results according to IntHout et al. [26]. Outlier and influence analyses were carried out following the recommendations of Harrer [24] and Viechtbauer and Cheung [27]. The small study effect was visually investigated on funnel plots. All statistical analyses were performed with the R [28] statistical environment and language, using the *meta* [29] and *dmetar* [24] packages.

#### 2.3.6. Statistical Analysis

In the study cohorts, both the Kaplan–Meier log-rank test and univariate Cox analysis were used for univariate analysis of OS. The nonparametric two-sided Wilcoxon rank sum test (Mann–Whitney test) was applied to compare the paired groups. In study cohort 2, nonparametric receiver operating characteristic (ROC) curves were used to determine the optimal cut-off value with the highest sensitivity and specificity for predicting patient death. In all tests, a p-value of at least 0.05 was considered significant. All statistical analyses were performed using IBM SPSS Statistics for Windows, version 27.0 (IBM Corp., Armonk, NY, USA).

## 3. Results

### 3.1. Patient Characteristics

In 2 independent cohorts, a total of 256 UBC patients underwent post hoc serum MMP-7 analysis. Patient and follow-up characteristics are shown in Table 1.

#### 3.1.1. Present Study Cohort 1

In cohort 1 of 188 UBC patients, the median age was 71 years (range: 21–90) and the median follow-up time was 24 months. Of the 188 patients, 56 had passed away since the last follow-up. At the time of the diagnosis, 80 (42.5%) patients had muscle-invasive UBC, of whom 32 (17%) were LN positive.

#### 3.1.2. Present Study Cohort 2

Cohort 2 included 68 patients with UBC in whom pretreatment (n = 68), postoperative serum MMP-7 levels (n = 57), and detailed postoperative LN status (n = 45) were measured. The median age was 66.4 years (range: 41–83), and the median follow-up time was 22.5 months. Of the 68 patients, 30 passed away at the last follow-up. At diagnosis, 65 (96%) patients had muscle-invasive urothelial cancer, of whom 30 (44%) were LN positive.

### 3.2. Correlation of MMP-7 Levels with Clinicopathological Parameters

In cohort 1, preoperative serum MMP-7 levels were not associated with gender or tumor grade. Significantly higher serum levels of MMP-7 were detected in patients over 65 years of age (*p* = 0.006) with muscle-invasive disease and LN metastases (*p* = 0.006 and *p* = 0.015) (Table 1).

**Figure 1:**
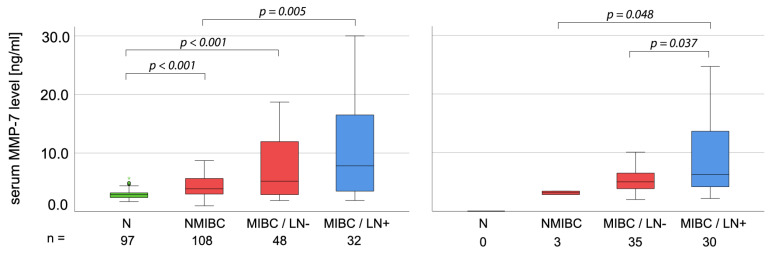
Boxplot of preoperative serum MMP-7 levels at various stages of UBC. *p*-values were calculated using the Wilcoxon Mann-Whitney U test. (NMIBC: non-muscle-invasive bladder cancer; MIBC: muscle-invasive bladder cancer).

In cohort 2, preoperative serum levels of MMP-7 showed no association with age or tumor grade. However, significantly higher serum levels of MMP-7 were found in patients with muscle-invasive disease and with LN metastases (*p* = 0.030, *p* = 0.021) (Table 1 and Figure 1).

### 3.3. Correlation of Clinicopathological Parameters and Pretreatment Serum MMP-7 Levels with Patient Prognosis

Results of the univariate OS analysis are shown in Table 2. The presence of high-grade, muscle-invasive tumors and metastases, as well as elevated serum MMP-7 levels before treatment, were associated with significantly shorter OS in the first study cohort (*p* = 0.001, *p* < 0.001 and *p* < 0.001, *p* < 0.001, respectively). Similarly, in cohort 2, patients with LN metastases and elevated pretreatment MMP-7 levels were associated with poorer OS (*p* = 0.002 and *p* < 0.001, Table 2).

**Figure 2:**
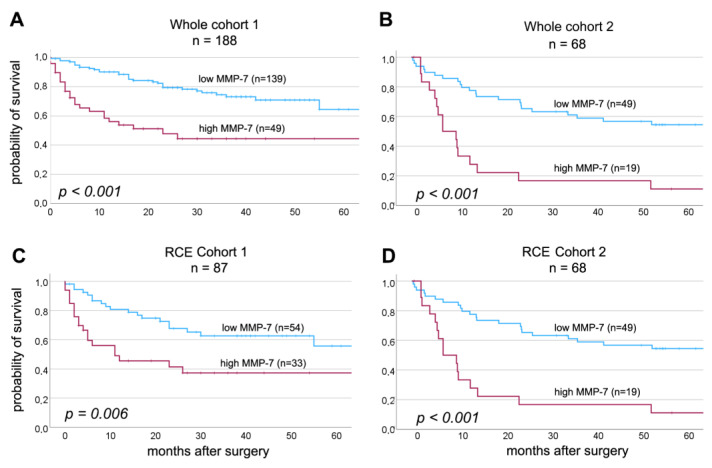
Overall survival curves in the whole cohorts ((**A**)—cohort 1; (**B**)—cohort 2) and in the subgroups of RC treated patients ((**C**)—cohort 1; (**D**)—cohort 2).

For patients who underwent RC, high serum MMP-7 levels were significantly associated with poorer OS (cohort 1: *p* = 0.006, cohort 2: *p* < 0.001, Table 2, Figure 2).

**Figure 3:**
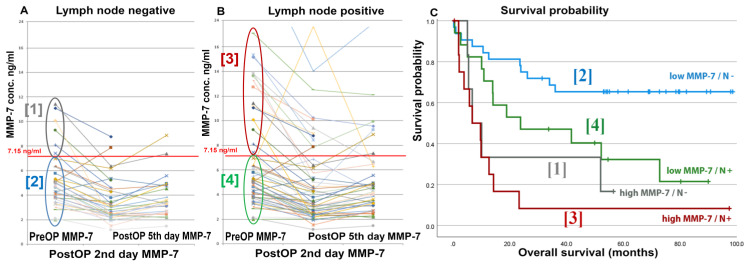
Pre- and postoperative (days 2 and 5) serum MMP-7 levels in LN negative (**A**) and positive patients (**B**) and corresponding Kaplan–Meier survival curves (**C**). Note that patients with high preoperative serum MMP-7 levels and LN-negative histological findings have comparatively poorer survival than those with LN-positive tumors.

In cohort 2, six patients had elevated pretreatment MMP-7 levels without LN metastases. This can be noted as false positivity, indicating metastases without metastases, but these patients had a similarly poor prognosis (2-year survival 30%) as those with LN positivity (30%) at the time of RC, in contrast to those with LN negativity and low MMP-7 levels (2-year survival 75%). This may suggest that patients with high pretreatment serum MMP-7 levels, but LN negativity, could have had positive LNs despite a failure to detect them during RC (Figure 3C).

A multivariate analysis of patients who underwent RC in cohort 1 revealed high pretreatment serum MMP-7 levels as an independent risk factor for shorter OS (*p* = 0.024; Table 3). These results were supported by the data from cohort 2, showing an independent prognostic value of high preoperative serum MMP-7 levels (*p*=0.004; Table 3).

### 3.4. Correlation of Pretreatment Serum MMP-7 Levels with the Localization of LN Metastases

To evaluate whether pretreatment MMP-7 levels could guide the decision between the regional vs. extended LND, in cohort 2, pretreatment serum MMP-7 levels were assessed in relation to LN metastasis pattern—MMP-7 levels correlated with the presence of metastases in LNs corresponding to the regional vs. extended LND. The diagnostic effectiveness of MMP-7 levels in predicting LN metastases at several cut-off values (Supplementary Figure S3) was determined by ROC curve analysis—two of the three cut-off values were 5.47 ng and 11.12 ng/mL based on the ROC curve analysis, and the third was 7.15 ng/mL, which was already used in a similar study [16]. A total of 68 patients had preoperative serum samples and information on overall LN status. The relationship between high and low levels of MMP-7 and LN positivity was assessed using the χ^2^ test (Supplementary Table S3) and a highly significant correlation was confirmed for all three cut-off values. At a cut-off of 5.47 ng/mL, approximately 70% of patients without LN metastases had low (<5.47 ng/mL) preoperative MMP-7 levels, identifying 63% of LN-positive cases. As the cut-off value increased, the number of false-positive cases decreased: six LN-negative cases had high MMP-7 levels at 7.15 ng/mL, and only one LN-negative case at 11.12 ng/mL, but the proportion of false-negative cases rose from 37% to 57% and finally to 63%, respectively. It is of utmost importance to find the balance between the most accurate diagnosis of LN-positive cases and minimizing false-negative cases. The optimal cut-off value seems to be 5.47 ng/mL, as at this cut-point, most patients with actual LN metastases can be classified as LN-positive based on high serum MMP-7 (63%) levels. However, even with this cut-off value, many (37%) patients with LN metastases would remain unidentified. Twenty-three patients had detailed information on the status of both regional and extended LNs. In the group of patients who underwent extended LND, a retrospective examination was conducted to determine whether an appropriate decision on the extent of LND could have been made based solely on the preoperative serum MMP-7 levels (Supplementary Table S4). Regional LND was performed in all cases, but appropriate decision-making had the greatest impact on patients who, despite having no positive regional LNs, had distant metastases (four of the examined patients, marked with yellow in Supplementary Table S4). It can be concluded that none of the examined cut-off values was particularly accurate in predicting the presence of positive LNs in the region of extended removal. When we used the value of 5.47 ng/mL, as a cut-off value, only one of the four patients with distant metastases had high pretreatment serum MMP-7 levels and would have been correctly selected for extended LND based on the MMP-7 levels alone. While for the two higher cut-off values, all four patients had low pretreatment serum MMP-7 levels, indicating only regional LND; distant metastases in these patients would have been missed (Supplementary Table S4).

### 3.5. Changes of MMP-7 Levels after Radical Cystectomy

For cohort 2, serum samples were available for 57 and 46 patients on postoperative days 2 and 5, respectively. Postoperatively, MMP-7 levels decreased drastically in the subgroup of patients who had high preoperative MMP-7 levels (Figure 3A). Despite the sharp increase in this subgroup, MMP-7 levels remained high in the LN-positive patients (Figure 3B), suggesting that tumor cells producing MMP-7 might remain behind after RC and LND. Serum MMP-7 levels at days 2 and 5 after RC were significantly associated with LN metastases (both *p* = 0.002) as well as with positive surgical margins (*p* = 0.003) (Supplementary Table S5). Postoperative MMP-7 levels were also associated with poorer patient survival (day 2: HR = 2.674, 95% CI: 1.136–6.293, *p* = 0.024; and day 5: HR = 3.185, 95% CI: 1.113–9.111, *p* = 0.031) (Supplementary Table S6). Moreover, changes in MMP-7 levels from RC to postoperative days 2 or 5 showed no significant correlation with OS.

### 3.6. Meta-Analysis of the Literature 

**Figure 4:**
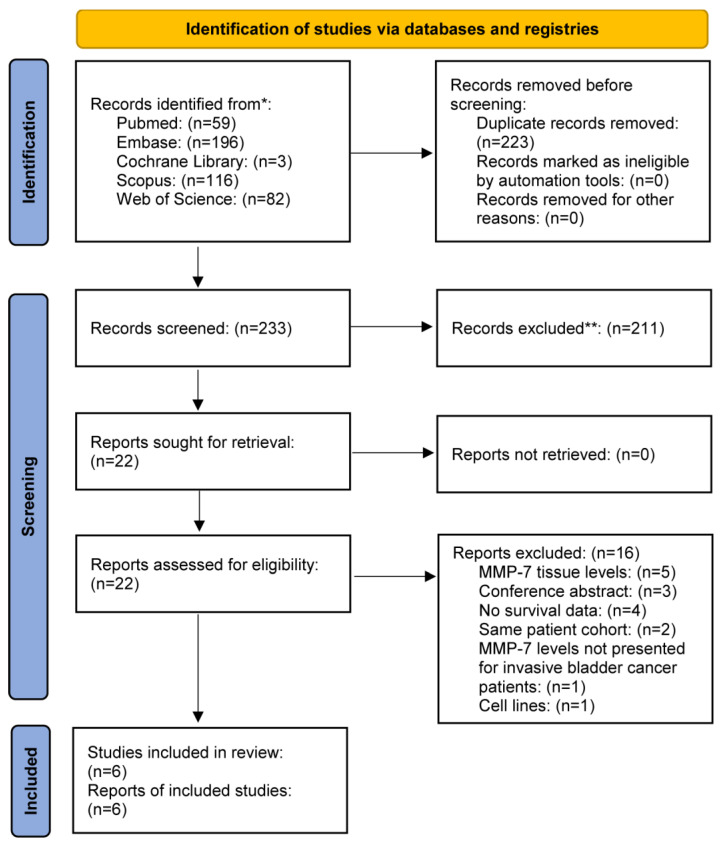
PRISMA 2020 flow diagram for new systematic reviews which included searches of databases, registries and other sources.

Our systematic search yielded six studies that were eligible for data extraction after retrieving 456 articles from the available databases (Figure 4). 

**Figure 5:**
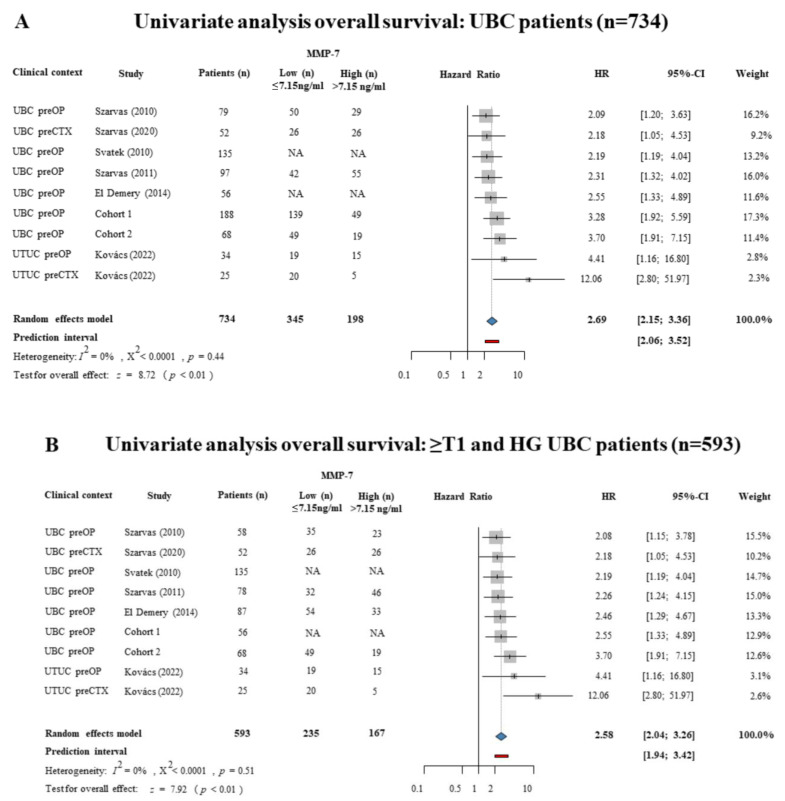

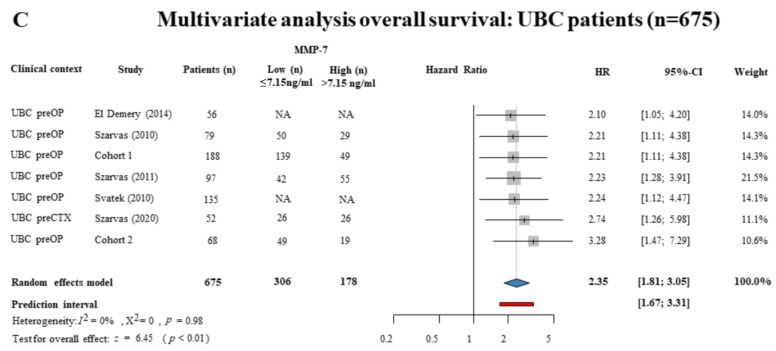
Forest plots representing hazard ratios of (**A**) all urothelial cancer patients (n = 734) OS, (**B**) hazard ratios of ≥T1 and HG urothelial cancer patients (n = 593) OS using the univariate Cox regression survival analysis, and (**C**) hazard ratios of urothelial carcinoma patients (n = 675) OS using multivariate Cox regression survival analysis in urothelial cancer patients with high pretreatment MMP-7 levels (cut-off value: 7.15 ng/mL) [16,21,30,31,32,33].

These were assessed together with the two patient cohorts (cohorts 1 and 2) presented above. Overall, eight urothelial cancer cohorts were included, with a total of 734 patients who had undergone surgery or systemic therapy and had pretreatment serum or plasma MMP-7 levels (Supplementary Table S2). Due to the high mortality of patients with muscle-invasive bladder cancer and the relatively homogenous population, the random-effects model was used (*p* = 0.44, I^2^ = 0%), and a pooled analysis showed a significant association with poorer OS in favor of patients with high pretreatment serum MMP-7 levels (HR = 2.69; 95% CI = 2.15–3.36) (Figure 5A). 

A subgroup analysis of 593 patients with muscle-invasive high-grade urothelial carcinoma who underwent radical surgery (RC, RNU) or chemotherapy (CTX) showed that high pretreatment MMP-7 levels were significantly correlated with reduced OS (HR = 2.58; 95% CI = 2.04–3.26) (Figure 5B). A multivariate analysis revealed, using the random effects model, that high serum MMP-7 levels were predictive of overall survival (HR = 2.35, 95% CI = 1.81–3.05) (Figure 5C).

## 4. Discussion

This combined post hoc cohort analysis and meta-analysis aimed to assess the prognostic significance of pretreatment MMP-7 levels in urothelial cancer patients undergoing surgical or systemic therapy. Additionally, we evaluated whether preoperative serum MMP-7 levels could support decision-making regarding the extent (extended vs. regional) of LND during radical cystectomy. Finally, we assessed postoperative MMP-7 levels to analyze dynamic changes and their prognostic values in UBC. Our results, in accordance with independent studies, consequently demonstrated that pretreatment serum and plasma MMP-7 levels were highly associated with shorter patient survival. We found significantly decreased serum MMP-7 levels after radical surgery. Pretreatment MMP-7 levels were not able to support the decision for extended, or rather regional, LND. Notably, the survival of patients with high preoperative MMP-7 levels but LN-negative histological results was poor, similar to that of those with histologically confirmed LN positivity.

Previous studies assessed the association between MMP-7 serum and plasma levels and outcomes in patients with urothelial cancer. The first study from 2010 on UBC demonstrated in a German patient cohort that elevated tissue expressions and serum concentrations of MMP-7 were associated with the presence of LNs and distant metastatic disease. The ROC analysis identified a serum concentration of 7.15 ng/mL as the cut-off value with the highest sensitivity (82%) and specificity (71%) for preoperative detection of LN positivity. Finally, high preoperative MMP-7 concentrations were independently associated with overall, disease-specific, and metastasis-free survival [16]. In a similar study, Svatek et al., using a multiplex, particle-based PRISMA cytometric assay to detect MMP-1, 2, 3, 7, 8, 9, and 12 in a U.S. cohort of patients with invasive UBC, found that elevated preoperative plasma MMP-7 levels were associated with significantly shorter time to cancer-related mortality [21]. A further study from Germany confirmed the prognostic significance of circulating plasma MMP-7 levels in predicting both overall and disease-specific survival [30]. El Demery et al. assessed a cohort of patients with advanced muscle-invasive UBC from France in 2014 and showed that high (≥11.5 ng/mL) serum MMP-7 concentrations were an independent prognostic factor for OS [31]. Benoit et al. from Switzerland found that serum MMP-7 levels were markedly elevated in muscle-invasive cancer compared to both non-muscle-invasive UBC and non-cancerous controls, suggesting that the use of these measurements might be useful in the selection of patients with a higher risk of more invasive or nodal-positive disease [34]. Bryan et al. assessed 422 proteins in serum samples from UBC patients from the UK and identified MMP-7 as one of the top five most significant prognostic serum biomarkers [35]. A further study demonstrated shorter OS in Hungarian UBC patients with elevated serum MMP-7 levels before platinum-based chemotherapy [32]. Kovács et al. found that preoperative serum MMP-7 levels were associated with shorter OS in Hungarian upper tract urothelial cancer patients, suggesting that serum MMP-7 may help to improve preoperative staging and prognosis in this disease [33].

In this study, we assessed the prognostic value of MMP-7 in two additional UBC cohorts. Cohort 1 included 188 patients with available preoperative (TURB or RC) serum samples and 97 non-tumorous controls. We found the lowest MMP-7 levels (2.9 ng/mL) in controls, followed by non-muscle-invasive UBC patients (3.9 ng/mL) and muscle-invasive UBC patients (5.3 ng/mL), with the highest levels in LN-positive patients (7.8 ng/mL). Accordingly, the OS of patients with high (>7.15 ng/mL) MMP-7 levels was significantly shorter in both the whole cohort and the subgroup of muscle-invasive UBC patients. These correlations remained significant in the multivariate analysis as well. 

In cohort 2 of 68 UBC patients who underwent RC and extended LND, we assessed the preoperative and postoperative (days 2 and 5) levels of MMP-7. Similarly, to our previous findings, higher serum MMP-7 levels were associated with LN positivity and shorter OS both in the univariate and multivariate analyses. In addition, we detected a significant decrease in serum MMP-7 levels in patients with high preoperative MMP-7 concentrations, suggesting tumor cells were the main source of serum MMP-7. Despite this reduction, postoperative MMP-7 levels remained higher than 7.15 ng/mL in most LN-positive cases (7/10), which may indicate the presence of residual tumors after surgery. Assessing MMP-7 levels in LN-positive and negative patients in cohorts 1 and 2, we found that only half of the patients (16/32 in cohort 1 and 17/30 in cohort 2) with histologically confirmed LN metastases had elevated preoperative MMP-7 levels (false negatives). This suggests that preoperative serum MMP-7 levels have a relatively high false-negative rate in the detection of LN metastases. Therefore, serum MMP-7 levels cannot be used to determine the extent of LND. On the other hand, only 15–20% (cohort 1: 33/153; cohort 2: 6/38) of histologically confirmed LN-negative patients had high MMP-7 levels. Notably, these technically false-positive patients (with high MMP-7 and histologically LN-negative tumors) had significantly worse prognoses compared to patients with low MMP-7 levels and LN-negative tumors. This suggests that the former patients might have had undetected (micro)metastases at RC and that MMP-7 may be able to identify these patients, who may therefore benefit from an early aggressive systemic therapy. Increased expression and serum concentrations of MMP-7 may support the formation of lymph node metastases by several molecular mechanisms such as cleaving cell adhesion molecules (e.g., E-cadherin), proapoptotic factors (Fas, FasL) [36], growth factors cytokines (TNF-α, TGF-β) and producing biologically active protein fragments (endostatin, angiostatin, RANKL) [37]. By these mechanisms, high MMP-7 expression is associated with reduced epithelial tissue integrity, increased cell motility, and invasive capacity, which may explain its association with a high risk of lymph node metastasis [38]. 

In addition to the post hoc serum analysis of the two UBC patient cohorts, we performed a systematic review and meta-analysis on the prognostic relevance of pretreatment circulating MMP-7 levels in urothelial cancer. Our systematic search identified six studies from four countries, which, together with our two cohorts, included an overall number of 734 patients. Furthermore, we also performed a subgroup analysis, including high-risk (≥T1 and HG) patients who underwent radical surgery. In both analyses, all included studies consequently showed a significant prognostic value for serum MMP-7 levels, with hazard ratios ranging from 2.08 to 12.06. The highest hazard ratios were observed in upper tract urothelial carcinomas, highlighting an even stronger prognostic relevance for MMP-7 in these patients. This may be of high clinical importance, considering that in upper tract urothelial carcinoma, the preoperative risk stratification is strongly limited by the inaccuracy of biopsies, leading to overtreatment in some patients and undertreatment in others. Our meta-analysis revealed a narrow prediction interval with reproducible results in independent studies. Based on them, currently available retrospective data sufficiently validate the use of the circulating pretreatment MMP-7 level as an independent prognostic factor in urothelial carcinoma, and therefore no further retrospective studies are needed. In addition, our metadata may provide a solid basis for calculating prospective study designs.

The above-described results and additional in vitro results suggest that MMP-7 is a promising molecular imaging and therapeutic target [39]. Recently, MMP-7 selective and specific molecular imaging methods have been developed, but these are still at the preliminary stage [40]. Furthermore, the detection of activated MMPs by radio-/fluorophore-labeled small molecule MMP inhibitors could be a clinically useful new tool to diagnose early (micro)metastases by molecular imaging techniques, such as SPECT and PET [41]. Previously performed large phase III clinical trials have failed to demonstrate the efficacy of broad-spectrum MMP inhibitors in cancer patients, which may be at least partly explained by the complex roles of MMPs both in physiological and pathological processes [42]. Therefore, subtype-specific MMP inhibitors may provide more targeted effects, and, therefore, their future therapeutic application remains promising. Despite the difficulties inherent in the high homology between members of the MMP family, synthetic MMP-7 selective/specific inhibitors have already been developed [43,44] and hold promise for future clinical application.

## 5. Conclusions

In conclusion, in our cohort study combined with a systematic review and meta-analysis, we synthesized the results of our own and previously published independent data and validated pretreatment serum MMP-7 as a prognostic factor in urothelial carcinoma. Independent studies have consequently found an approximately 2.5-fold risk of mortality in patients with high MMP-7 levels. Our data demonstrated that preoperative MMP-7 levels cannot be used to decide on the extent of LND. On the other hand, high preoperative MMP-7 levels may identify patients with undetected (micro)metastases. These patients are at high risk of disease progression and would most probably benefit from early aggressive therapy. Serum MMP-7 levels can be considered a validated preoperative prognostic serum marker in urothelial cancer and may therefore help to optimize therapeutic decision-making. Finally, MMP-7 imaging and targeted therapy hold promise for future diagnostic and therapeutic strategies.

## Figures and Tables

**Table 1:** Patient characteristics and their correlation with preoperative MMP-7 levels.

		Cohort 1			Cohort 2		
		PreOP Serum MMP-7 cc.			PreOP Serum MMP-7 cc.		
Parameters		n	Median (Range)	*p*	n	Median (Range)	*p*
Whole UBC cohort		188	4.2 (1.0–75.2)	<0.001	68	5.21 (1.99–24.71)	-
Non-tumorous control		97	2.9 (1.7–5.7)	-	0	-	-
Age	≤65	51	3.6 (1.6–75.2)	0.006	28	5.26 (1.99–15.34)	0.866
	>65	137	4.6 (1.9–62.0)	-	40	5.20 (2.09–24.70)	-
Sex	male	149	4.1 (1.6–62.0)	0.622	43	4.77 (2.09–24.00)	0.239
	female	39	4.4 (1.0–75.2)	-	25	6.11 (2.09–24.70)	-
Stage	Ta	81	3.9 (1.4–18.2)	-	0	-	-
	Cis	8	3.6 (2.4–2.6)	-	2	3.13 (2.83–3.43)	-
	T1	19	3.9 (1.0–15.9)	-	1	3.62	-
	T2	28	5.8 (1.9–75.2)	-	20	4.87 (1.99–9.28)	-
	T3	27	4.4 (2.3–26.2)	-	32	6.23 (2.98–24.71)	-
	T4	25	6.2 (1.9–62.0)	-	13	5.91 (2.09–17.09)	-
Non-inv.	(Cis-Ta-T1)	108	3.9 (1.0–18.2)	0.006	3	3.43 (2.82–3.62)	0.030
Invasive	(T2–T4)	80	5.3 (1.9–75.2)	-	65	5.31 (1.99–24.71)	
Grade	G1	37	4.1 (1.9–18.0)	-	0	-	-
	G2	93	4.4 (1.0–30.0)	-	9	6.11 (2.83–12.73)	-
	G3	58	4.1 (1.9–75.2)	-	47	5.31 (2.09–24.71)	-
	Unknown	0		-	12		-
Low-grade	(G1–2)	130	4.3 (1.0–30.0)	0.622	9	6.11 (2.83–12.73)	0.973
High-grade	(G3)	58	4.1 (1.9–75.2)	-	47	5.31 (2.09–24.71)	
Surgery	TURB	101	3.9 (1.0–18.2)	0.021	0	-	-
	RC	87	4.7 (1.9–75.2)	-	68	5.21 (1.99–24.71)	-
Lymph node	N0/Nx	156	4.0 (1.0–75.2)	0.015	38	4.87 (1.99–24.00)	0.021
	N+	32	7.8 (1.9–75.2)		30	6.25 (2.17–24.71)	

**Table 2:** Impact of clinicopathological parameters and preoperative serum MMP-7 levels on OS in cohorts 1 and 2. (Cis: carcinoma in situ; PreOP: preoperative).

				Cohort 1			Cohort 2		
				OS			OS		
General Data		n	HR	95% CI	*p*	n	HR	95% CI	*p*
Age	≤65	51	ref.			28	ref.		
	>65	137	1.727	0.892–3.343	0.105	40	1.444	0.757–2.756	0.265
Sex	female	39	ref.			43	ref.		
	male	149	0.660	0.369–1.179	0.160	25	0.902	0.463–1.758	0.762
Stage	NMIBC (Cis-Ta-T1)	108	ref.				ref.		
	MIBC (T2–T4)	80	3.705	2.093–6.558	<0.001	65	2.085	0.286–15.206	0.469
Grade	Low-grade (G1–2)	130	ref.			9	ref.		
	High-grade (G3)	58	2.489	1.470–4.212	0.001	47	1.389	0.541–3.568	0.495
LN status	N0/Nx	156	ref.			38	ref.		
	N+	32	5.523	3.213–9.492	<0.001	30	2.721	1.427–5.189	0.002
PreOP MMP-7 whole cohort		188				68			
	≤7.15 ng/mL	139	ref.			49	ref.		
	>7.15 ng/mL	49	3.276	1.921–5.589	<0.001	19	3.699	1.913–7.153	<0.001
NMIBC (Ta-Cis-T1)		108				3			
	≤7.15 ng/mL	92	ref.			3	-		
	>7.15 ng/mL	16	2.743	0.885–8.502	0.080	0	-	-	-
MIBC (T2–T4)		80				65			
	≤7.15 ng/mL	47	ref.			46	ref.		
	>7.15 ng/mL	33	2.217	1.176–4.180	0.014	19	3.623	1.860–7.055	<0.001
RC		87				68			
	≤7.15 ng/mL	54	ref.			48	ref.		
	>7.15 ng/mL	33	2.455	1.291–4.669	0.006	19	3.699	1.913–7.153	<0.001
TURB		101				0			
	≤7.15 ng/mL	85	ref.			0	-		
	>7.15 ng/mL	16	3.085	1.090–8.736	0.034	0	-	-	-

**Table 3:** Multivariate overall survival analysis.

		Cohort 1			Cohort 2		
		OS			OS		
General Data		HR	95% CI	*p*	HR	95% CI	*p*
Stage	≤T2 vs. >T2	4.253	1.764–10.252	0.001	1.272	0.493–3.281	0.619
Grade	2 vs. 3	1.424	0.664–3.054	0.364	1.256	0.484–3.261	0.640
Lymph node	neg. vs. pos.	2.316	1.115–4.810	0.024	1.434	0.685–2.999	0.339
MMP-7: ≤7.15 vs. >7.15 ng/mL		2.206	1.111–4.380	0.024	3.279	1.475–7.290	0.004

## Data Availability

The data presented in this study are available on request from the corresponding author.

## Supplementary Materials

The following supporting information can be downloaded at: https://www.mdpi.com/article/10.3390/ijms24097859/s1.
